# Non-Destructive Evaluation of Damage and Electricity Characteristics in 4H-SiC Induced by Ion Irradiation via Raman Spectroscopy

**DOI:** 10.3390/ma18215057

**Published:** 2025-11-06

**Authors:** Hui Dai, Zhiyan Hou, Xinqing Han, Jiacheng Liang, Anxin Jiao, Zhixian Wei, Chen Wu, Ke Sun, Yong Liu, Xuelin Wang

**Affiliations:** 1 Key Laboratory of Particle Physics and Particle Irradiation (MOE), Institute of Frontier and Interdisciplinary Science, Shandong University, Qingdao 266237, China; 202221087@mail.sdu.edu.cn (H.D.); 202316938@mail.sdu.edu.cn (Z.H.); weizxlf@sdu.edu.cn (Z.W.); 2 Shandong Provincial Key Laboratory of Nuclear Science, Nuclear Energy Technology and Comprehensive Utilization, School of Nuclear Science, Energy and Power Engineering, Shandong University, Jinan 250061, China; xqhan@sdu.edu.cn (X.H.); 202494000029@sdu.edu.cn (C.W.); sunkeke@sdu.edu.cn (K.S.); 3 School of Physics and Optoelectronics Engineering, Ludong University, Yantai 264025, China; ljc@ldu.edu.cn (J.L.); jiaoax@ldu.edu.cn (A.J.)

**Keywords:** 4H-SiC, ion irradiation, Raman spectroscopy, irradiated damage, conductivity degradation

## Abstract

To optimize the design of silicon carbide (SiC) devices for applications in space and nuclear environments, this work introduces varying degrees of lattice damage into SiC through controlled irradiation conditions, with Raman spectroscopy revealing its damage evolution behavior and quantitatively characterizing the increasing trend of disorder with irradiation fluences. Fine analysis of the A_1_(LO) phonon mode demonstrates that proliferation of irradiation-induced acceptor centers and accumulation of scattering defects lead to significant attenuation of carrier concentration and mobility (cross-verified by Hall effect measurements), thereby causing degradation in electrical conductivity of SiC. Subsequent electrical testing confirms an orders-of-magnitude reduction in conductivity, establishing a quantitative correlation model with total disorder quantified by the DI/DS model. The non-destructive Raman technique enables simultaneous acquisition of material damage characteristics and quantitative electrical performance degradation, providing a predictive framework for the evolution of electrical behavior for SiC under irradiation damage, with significant implications for optimizing irradiation-hardened device designs.

## 1. Introduction

Silicon carbide (SiC) features a wider band gap, higher critical electric field, approximately twice the saturation velocity, and three times the thermal conductivity of silicon. It also exhibits a higher average displacement energy [1]. These superior physical and electronic properties render SiC not only a strong candidate to replace silicon in power and high-frequency applications, but also particularly suitable for use in extreme environments characterized by intense radiation [2]. Spaceborne systems, for example, are routinely exposed to ionizing radiation such as gamma rays, as well as heavy and light-charged particles including protons and high-energy electrons [3,4]. Likewise, devices deployed in Generation IV nuclear reactors must withstand fast and thermal neutrons and high-energy gamma and beta radiation [5]. Compared to other semiconductor materials, SiC offers an optimal combination of properties to endure such harsh conditions [6]. Therefore, to effectively design and deploy SiC-based devices in harsh environments, it is essential to understand the mechanisms and quantitative relationships between radiation damage and electrical properties, thereby enhancing their radiation resistance. Ion irradiation offers several advantages, including operational simplicity, high flexibility, and ease of implementation [7]. Its energy and fluence can be precisely controlled, enabling tailored modification of material microstructures [8]. It plays a crucial role in advancing both fundamental research and applied understanding of radiation-induced effects [9,10,11,12,13].

Conventional techniques such as Rutherford backscattering spectroscopy in channeling mode (RBS/C) and transmission electron microscopy (TEM) are commonly employed to investigate structural damage in materials [14,15]; however, both are inherently destructive. In contrast, Raman spectroscopy offers a minimally invasive alternative that yields valuable structural insights. It is particularly well-suited for characterizing silicon carbide due to its non-destructive nature and the absence of special sample preparation requirements [16]. Moreover, the strong covalent bonding in SiC leads to high Raman scattering efficiency, enabling the acquisition of strong, well-defined spectral signals [17,18]. Key spectral parameters—including intensity, peak width, frequency shift, and polarization—can provide detailed information regarding the crystal quality and structural integrity of the material. Raman spectroscopy has been extensively utilized in a variety of applications, including the measurement of residual stress in thin films [19], analysis of impurities and defects [20], and investigation of electronic excitations in semiconductor materials [21]. A notable advantage of this technique lies in its capability to probe the electrical properties of polar semiconductors [22]. Specifically, it enables the detection of longitudinal optical phonon–plasmon coupling (LOPC) modes, whose spectral characteristics, such as frequency, linewidth, and intensity, are strongly dependent on free carrier concentration and damping effects. Unlike conventional electrical characterization methods, Raman spectroscopy does not require the use of electrodes and imposes no stringent requirements on sample preparation. Owing to these advantages, numerous researchers have employed this approach to study the electrical behavior of various semiconductor systems. For instance, Harima H. et al. investigated the Raman spectra of longitudinal optical phonon–plasmon coupling (LOPC) modes in n-type 6H-SiC and 4H-SiC bulk crystals with varying carrier concentrations. Their results revealed that 6H-SiC exhibits greater anisotropy in carrier mobility compared to 4H-SiC [23]. Chafai M. et al. performed a theoretical analysis of the LOPC modes in n-type 4H-SiC and established a quantitative relationship between the LOPC frequency (*ω_LOPC_*) and the free carrier concentration, based on the plasmon frequency (*ω_p_*) and plasmon damping constant (*γ_p_*) [24]. Yang S. et al. combined electron backscattering diffraction and confocal Raman microscopy to meticulously map the strain distribution in He^+^ ion-irradiated 4H-SiC, revealing the propagation of strain into the unirradiated substrate and its correlation with carrier density variations [25]. In another study, Zhang L. et al. employed Raman scattering spectroscopy to examine GaN films irradiated with 290 MeV ^238^U^32+^ ions and observed a significant reduction in free carrier concentration, which they attributed to the formation of nitrogen and gallium vacancies [26].

This study aims to investigate the evolution of electrical properties in 4H-SiC before and after ion irradiation using non-destructive Raman spectroscopy, complemented by Hall effect and conductivity measurements to overcome the limitations of relying solely on optical methods. Bulk 4H-SiC samples were irradiated with O and Au ions. Changes in electrical performance were evaluated through current–voltage (I-V) measurements, while variations in free carrier concentration and mobility were quantitatively determined via Raman spectral analysis and Hall effect measurements. Furthermore, we innovatively established a quantitative model that correlates total disorder with electrical properties, providing a predictive framework for evaluating the evolution of electrical characteristics of SiC under irradiation damage.

## 2. Materials and Methods

Optically polished 4H-SiC crystals (10 × 10 × 0.5 mm^3^) with <0001> orientation were prepared by Metal–organic Chemical Vapor Deposition (MOCVD) and commercially purchased from Shanghai Institute of Optics and Fine Mechanics, Shanghai, China. The samples were irradiated with 2 MeV O and 3 MeV Au ions at 300 K using the 2 × 1.7 MeV tandem electrostatic accelerator at the State Key Laboratory of Nuclear Physics and Technology, Peking University. O ions, due to their lower mass, induce relatively mild damage, whereas Au ions, being significantly heavier, generate more extensive damage. The combined use of both ions allows for a broad and tunable range of irradiation damage, meeting the requirements of this study. All irradiation experiments were conducted in a vacuum chamber with a base pressure of approximately 2 × 10^−6^ mbar. To avoid channeling effects during irradiation, the ion beam was scanned at a 7° angle relative to the sample surface normal. It is well established that high-flux ion irradiation can lead to significant macroscopic heating of materials. The macroscopic heating of the samples under ion irradiation is not discussed in the present work, but low beam fluxes (5.0 × 10^10^ cm^−2^s^−1^ for O ions, 8.3 × 10^10^ cm^−2^s^−1^ for Au ions) were used in order to avoid any undesired ion beam annealing and charge accumulation on the samples during the irradiation.

**Figure 1:**
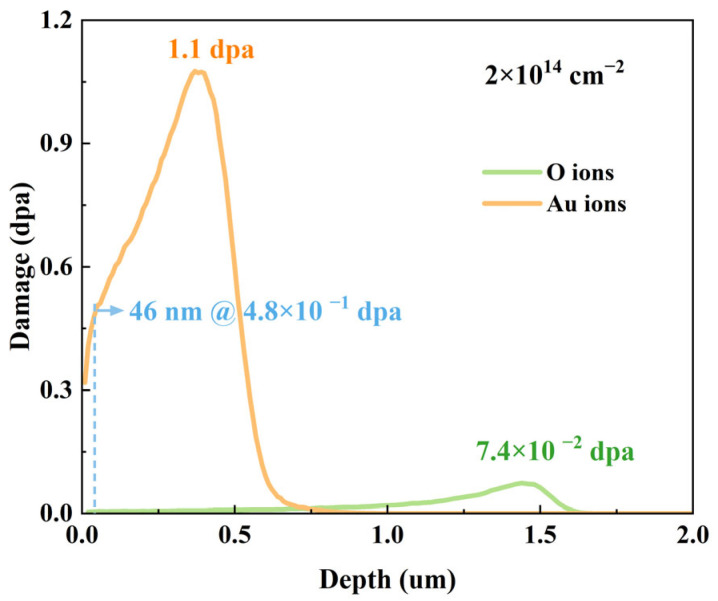
SRIM-simulated dpa depth profiles induced by 2 MeV O ions and 3 MeV Au ions at fluence of 2 × 10^14^ cm^−2^.

The depth profiles of displacements per atom (dpa) induced by energetic ion irradiation in 4H-SiC were simulated using the Stopping and Range of Ions in Matter (SRIM) 2013 software with full-cascade mode. A density of 3.21 g/cm^3^ was used for 4H-SiC, with threshold displacement energies of 35 eV for Si and 20 eV for C [27]. The SRIM-simulated dpa depth profiles under ion irradiation are shown in Figure 1.

Raman spectra were acquired using a confocal Raman spectrometer (LabRAM HR Evolution, Horiba Scientific, Kyoto, Japan), equipped with an xyz automated stage with an accuracy of 0.1 μm. The measurements were performed in backscattering mode using a solid-state laser with a wavelength of 532 nm. The confocal slit diameter was set to 50 μm, providing a spectral resolution of approximately 0.35 cm^−1^. To comprehensively capture the vibrational characteristics of SiC chemical bonds after irradiation, the spectral range was set from 100 to 2000 cm^−1^. Measurements were performed with a grating of 1800 grooves/mm, a laser power of 10 mW, an integration time of 15 s, and a 100× objective lens (NA = 0.9), resulting in a laser spot diameter of approximately 1 μm. Each sample spectrum was collected three times for statistical reliability. Under these conditions, the selected laser power and short integration time do not produce measurable thermal shifts or laser-induced damage in pristine 4H-SiC, ensuring that the observed spectral variations can be exclusively attributed to ion irradiation effects. The 532 nm excitation light has a penetration depth of approximately 2 mm in 4H-SiC; however, due to the confocal configuration, only signals from a depth of about 2 μm reach the detector [5]. Therefore, the irradiated regions fall within the detection depth. However, for amorphous SiC, the detection depth is limited to approximately 46 nm [17], meaning the Raman analysis is localized to the near-surface region.

Hall effect measurements were performed using an East Changing ET-9000 system (East Changing Technologies, Beijing, China), and the carrier concentration and mobility were measured at room temperature.

Metal–semiconductor–metal (MSM) structured devices were fabricated by thermally evaporating platinum electrodes onto 4H-SiC crystals. Electrical measurements were conducted at room temperature using an Agilent B1500A semiconductor parameter analyzer (Agilent Technologies, Santa Clara, CA, USA) connected to a probe station.

## 3. Results and Discussion

### 3.1. Raman Spectra of 4H-SiC Irradiated with O and Au Ions

**Figure 2:**
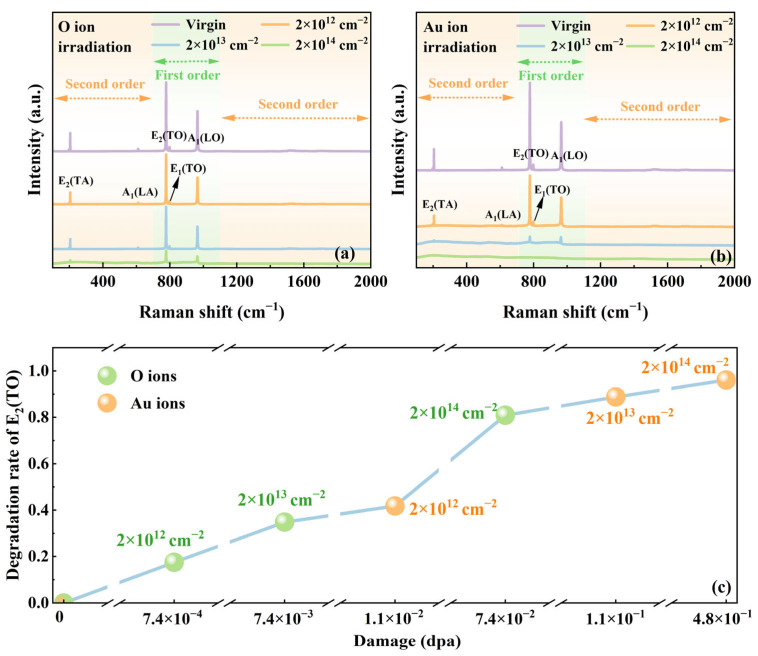
Raman spectra of 4H-SiC irradiated with (**a**) 2 MeV O ions and (**b**) 3 MeV Au ions at varying fluences. (**c**) Degradation rate of the E_2_(TO) phonon mode as a function of irradiation damage for O and Au ions.

Among the 24 lattice modes of 4H-SiC, only 10 modes (3A_1_ + 3E_1_ + 4E_2_) exhibit Raman activity. In the Raman spectrum of SiC, the 700–1100 cm^−1^ range is assigned to the first order, with the 100–700 cm^−1^ and 1100–2000 cm^−1^ regions corresponding to the second order. The characteristic vibrational regions are divided into three distinct regions: the 20–600 cm^−1^ region is assigned to vibrational modes primarily associated with atomic displacements in Si-Si bonds, the 600–1000 cm^−1^ region to modes involving Si-C bonds, and 1000–1800 cm^−1^ regions to modes involving C-C bonds [28]. The Raman spectra of 4H-SiC irradiated with O and Au ions were systematically characterized, with the corresponding results presented in Figure 2a,b, respectively. The unirradiated 4H-SiC exhibits five distinct Raman modes below 1200 cm^−1^: transverse acoustic phonon E_2_(TA) at 204.8 cm^−1^, longitudinal acoustic phonon A_1_(LA) at 610.2 cm^−1^, transverse optical phonon E_2_(TO) at 777.4 cm^−1^, transverse optical mode E_1_(TO) at 799.1 cm^−1^, and longitudinal optical phonon A_1_(LO) at 965.1 cm^−1^. The position and shape of A_1_(LO) are related to the carrier concentration, and its vibrational mode results from the coupling between carriers and phonons. Additionally, the main second-order Raman scattering peaks are observed at 1517.4 and 1708.5 cm^−1^. Notably, the E_2_(TO) mode, associated with crystal defects, dominates the 4H-SiC Raman spectrum and serves as a key indicator of lattice integrity [29,30]. The degradation rate of the E_2_(TO) mode intensity as a function of irradiation damage (dpa) is shown in Figure 2c. Compared to the unirradiated sample, the E_2_(TO) peak intensity of the irradiated samples decreases, with a progressive reduction observed as dpa value increases. Under the same irradiation fluence, samples irradiated with Au ions exhibit a more pronounced reduction in Raman characteristic peak intensity than samples irradiated with O ions, which is attributed to greater lattice damage induced by the heavier mass of Au ions.

**Figure 3:**
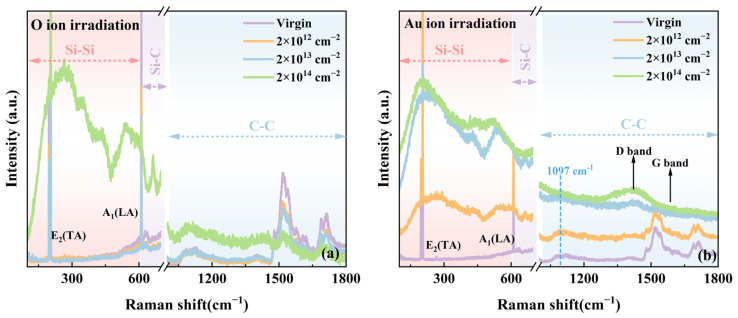
Second-order Raman spectra of 4H-SiC irradiated with different fluences: (**a**) 2 MeV O ions, (**b**) 3 MeV Au ions.

To facilitate clearer analysis of the weaker Raman peaks in the second-order spectral region, the first-order spectra in Figure 3 have been deliberately excluded. Notably, at a fluence of 2 × 10^14^ cm^−2^ under O ion irradiation, several new Raman peaks appeared in the 200–600 cm^−1^ range of the SiC Raman spectrum. These peaks are associated with the phonon vibration modes of Si-Si bonds. Specifically, the peaks at approximately 260 cm^−1^ and 347 cm^−1^ correspond to the LA and LO modes in crystalline silicon [28]. The peak at approximately 435 cm^−1^ is attributed to Si-O bond vibrations [28]. The peak near 540 cm^−1^ corresponds to the transverse optical (TO) mode of crystalline silicon [31]. The spectral region from 1000 to 1800 cm^−1^ corresponds to the phonon vibration modes of C-C bonds. Specifically, the peak at around 1097 cm^−1^ is associated with the sp^3^ hybridized C-C bond vibration, while the peak near 1410 cm^−1^ corresponds to the sp^2^ hybridized C-C bond vibration, also known as the D band. The Au ion irradiation is shown in Figure 3b; new Raman peaks corresponding to Si-Si phonon vibration modes similar to those irradiated by O ions appear in the 200–600 cm^−1^ range under all irradiation conditions. These vibrations are most prominent at a fluence of 2 × 10^14^ cm^−2^. Within the C-C vibration region (1000–1800 cm^−1^), the intensity of the peak near 1097 cm^−1^ increases as the irradiation fluence increases from low to high levels. In addition, the D band near 1410 cm^−1^ becomes more prominent with increasing fluence. The appearance of new Raman peaks in both the Si-Si and C-C vibration regions indicates the formation of heteronuclear chemical bonds in the damaged regions of 4H-SiC irradiated with O and Au ions. In summary, as the irradiation fluence increases, new peaks first emerge in the Si-Si vibration region, followed by peaks in the C-C vibration region. The underlying reasons for this phenomenon are twofold: (1) The formation energy of Si-Si bonds (221.8 kJ/mol) is significantly lower than that of C-C bonds (347.3 kJ/mol), making Si-Si bonding more thermodynamically favorable [32]. (2) Bond length also plays a crucial role: the Si-Si bond length (0.23 nm) is longer than that of the C-C bond (0.15 nm) [33,34]. Radiation-induced lattice swelling in 4H-SiC preferentially promotes the formation of Si-Si bonds over C-C bonds. Therefore, with increasing fluence and intensified lattice damage, the expanded atomic spacing progressively decreases, allowing C-C bonds to form after the initial formation of Si-Si bonds.

### 3.2. Relative Disorder of 4H-SiC Irradiated with O and Au Ions

**Figure 4:**
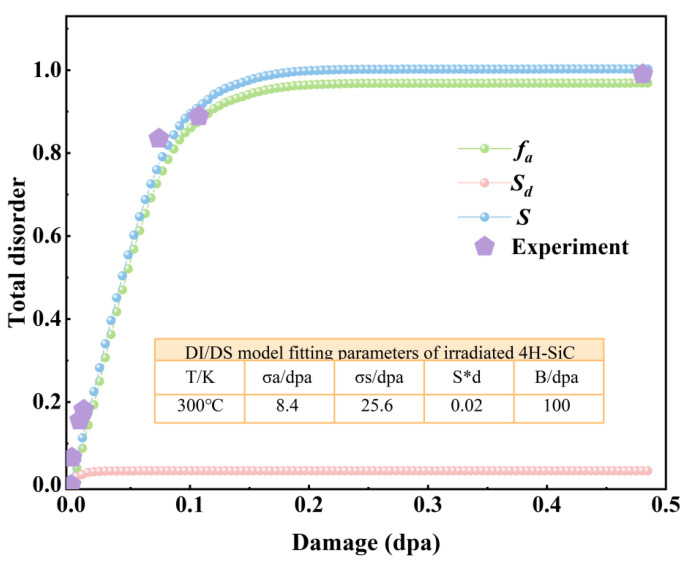
Relative disorder as a function of the local dpa, and fitted damage accumulation curve for 4H-SiC irradiated with 3 MeV Au ions and 2 MeV O ions.

According to the methodology established by Menzel et al. [35], the total disorder degree is quantified based on the integrated area of the first-order Raman peaks and is defined as follows:

(1) $$S=1-\frac{A}{A_{Virgin}}$$

where *A* and *A_Virgin_* represent the integrated areas of the first-order Raman peaks for irradiated and pristine 4H-SiC, respectively. Figure 4 shows the variation in total disorder as a function of fluence for 4H-SiC irradiated with O and Au ions. Differences in recrystallization behavior between Au and O ion irradiation are not considered; therefore, the data from both ion species are combined to represent the relationship between total disorder and irradiation damage. As shown in Figure 4, the total disorder increases with irradiation damage and gradually approaches saturation (*S* = 1). The dotted line in Figure 4 represents the fitting result of the DI/DS model [34], where the total disorder S is defined as

(2) $$S=f_{a}+S_{d}+S_{c}$$

where *f_a_* represents the disorder introduced by irradiation-induced amorphization, *S_d_* denotes the disorder caused by interstitial atoms and interstitial clusters within the crystal, and *S_c_* refers to extended defects formed at high temperatures. The amorphization-induced disorder component can be described by Equation (3).

(3) $$f_{a}=1-\left(\sigma_{a}+\sigma_{s}\right)/\left(\sigma_{s}+\sigma_{a}\exp\left[\left(\sigma_{a}+\sigma_{s}\right)D\right]\right)$$

where *σ_a_* is the amorphization cross-section due to direct collisions; *σ_s_* represents the effective cross-section for amorphization caused by defects; and *D* is the corresponding irradiation fluence (dpa). The point defects generated by irradiation and the disorder introduced by clusters are modeled using a simple point defect approach, as expressed in Equation (4).

(4) $$S_{d}=S_{d}^{*}\left[1-\exp\left(-BD\right)\right]\left(1-f_{a}\right)$$

where S*_d_ represents the saturation value of disorder induced by defects along the specified direction, which is proportional to the corresponding fluence; *B* is proportional to the volume of effective recombination of these defects. The accumulation of disorder due to extended defect clusters formed at high temperatures follows a pattern similar to point defect generation, as expressed in Equation (5).

(5) $$S_{c}=S_{c}^{*}\left[1-\exp\left(-RD\right)\right]\left(1-f_{a}\right)$$

where S*_c_ is the saturation value of disorder caused by the formation of extended defect clusters; *R* is proportional to the strength of the effective absorption well that forms the extended defect clusters. The DI/DS model fitting parameters of 4H-SiC irradiated by Au and O ions are listed in the table embedded in Figure 4. During the fitting process, the fitting values given by Zhang et al. [36] and Jiang et al. [37] were referenced. Regarding *S_d_*, it is assumed that its contribution to the total disorder results from the accumulation of defects in the form of point defects or small clusters. Its saturation value (~0.02) is approximately equal to the saturation value (~0.025) obtained through fitting based on Rutherford backscattering spectrometry (RBS/C) [38]. Furthermore, *σ_s_* is significantly larger than *σ_a_*, which aligns with previous reports [39,40]. These observations suggest that the probability of direct amorphization of SiC is very low, and that defect-stimulated amorphization is the dominant mechanism in SiC [41,42].

For 4H-SiC irradiated by O and Au ions, *f_a_* is much larger than *S_d_* in all irradiation fluence ranges; *f_a_* contributes more to the total disorder than *S_d_*, as reported in previous studies on SiC [37], indicating that the disorder in 4H-SiC is primarily induced by irradiation-induced amorphization. The total disorder *S* tends to saturate when the irradiation fluence exceeds 0.11 dpa, which is close to the amorphous critical value obtained through fitting based on RBS/C [37], indicating that 4H-SiC has become completely amorphous.

### 3.3. Carrier Concentration and Mobility of 4H-SiC Irradiated with O and Au Ions

**Figure 5:**
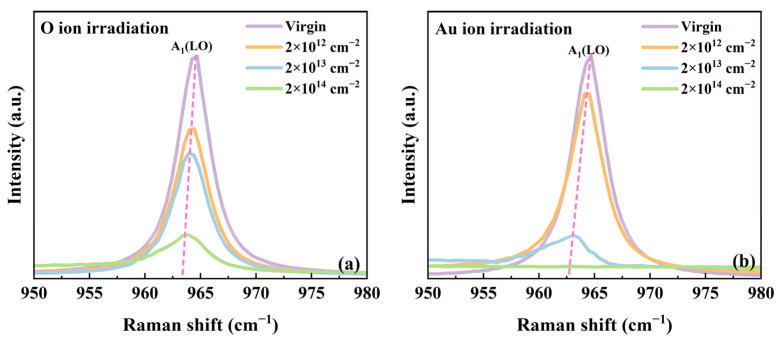
The Raman spectra corresponding to A_1_(LO) mode in 4H-SiC irradiated with (**a**) 2 MeV O ions and (**b**) 3 MeV Au ions.

To investigate the evolution of carrier concentration and mobility induced by irradiation, an in-depth analysis of the Raman spectra was performed. Figure 5a,b show the Raman spectral region corresponding to the A_1_(LO) mode of irradiated 4H-SiC. A pronounced redshift of the A_1_(LO) peak is observed in 4H-SiC subjected to O and Au ion irradiation. At the highest irradiation fluence, the A_1_(LO) mode shows a redshift of 1.8 cm^−1^ for Au ions and 1.2 cm^−1^ for O ions, together with a asymmetry in the line shape. In polar semiconductors, the collective excitation of free carriers (plasmons) interacts with longitudinal optical (LO) phonons via macroscopic electric fields, forming longitudinal optical phonon–plasmon coupling (LOPC) modes. The Raman line shape of the LOPC mode is highly sensitive to free carrier concentration. Therefore, the free carrier concentration (*n*) and carrier mobility (*μ*) in SiC can be extracted by analyzing the Raman line shape of the LOPC mode. According to the theoretical fitting established by W. L. Faust [43] and G. Irmer [44] in the framework of the dielectric model, the Raman intensity of the LOPC mode can be described as

(6) IA=d2SdϖdΩA=SAϖIm−1εA(ϖ)=1+2Cϖt2Δϖp2γϖt2−ϖ2−ϖ2Γϖ2+γ2−ϖp2+C2ϖt4Δϖl2−ϖt2ϖp2γϖl2−ϖt2+Γϖp2−2ϖ2+ϖ2Γϖ2+γ2Δ=ϖp2γϖt2−ϖ22+ϖΓ2+ϖ2Γϖl2−ϖt2ϖ2+γ2εϖ=ε∞1+ϖl2−ϖt2ϖt2−ϖ2−iϖΓ−ϖp2ϖϖ+iγ

(7) $$\varpi_{p}^{2}=\frac{4\pi ne^{2}}{\varepsilon_{\infty }m^{*}}$$

(8) $$\gamma =\frac{e}{m^{*}\mu }$$

where, for 4H-SiC, *ω_l_* and *ω_t_* are the frequencies of TO and LO phonons, *ω_l_* = 964.08 cm^−1^ and *ω_t_* = 776.95 cm^−1^. *ε* is the dielectric function, and *ε_∞_* is the high-frequency dielectric constant, the value of which is 6.78. *e* is the electron charge, *γ* is the damping constant of the plasma, *τ* is the damping constant of the phonon, *ω_p_* is the frequency of the plasma, *n* is the free carrier concentration, and m* is the effective mass of the free carrier, which is equal to 0.48 *m_e_*. *μ* is the free carrier mobility, *ω* is the Raman frequency shift, and *C* is the Faust–Henry coefficient, generally 0.43. *C* and *S* are regarded as constant coefficients.

**Figure 6:**
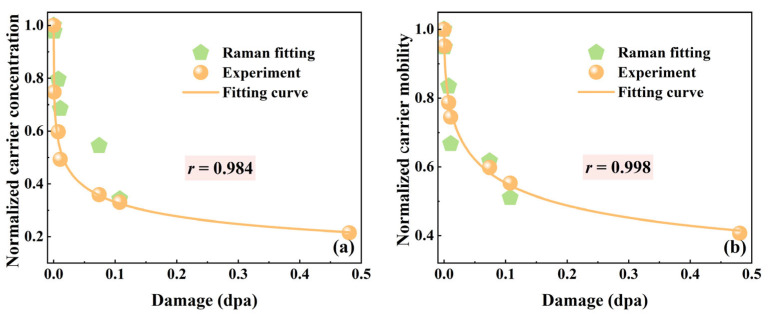
(**a**) Normalized carrier concentration. (**b**) Normalized carrier mobility as functions of fluence, parameterized by the Pearson correlation coefficient (r).

The experimental curves were fitted using the adjustable parameters *ω_p_*, *γ*, and *τ* in Equation (6). The electron effective mass (m*), constant (*C*) and high-frequency dielectric constant (*ε_∞_*) under 532 nm laser excitation were taken from Reference [23]. The LOPC peaks of all samples were fitted using the same analytical procedure. The values of carrier concentration (*n*) and mobility (*μ*) were calculated from the fitted parameters *ω_p_* and *γ* using Equations (7) and (8), respectively. The resulting variations in carrier concentration and mobility as a function of irradiation damage (dpa) are shown in Figure 6a,b. As shown in Figure 6, the carrier concentration of 4H-SiC decreases with increasing dpa. This is attributed to the interaction between incident ions and the SiC lattice. When ions penetrate the SiC crystal, they collide with lattice atoms, generating numerous point defects. As the irradiation fluence increases, these defects accumulate and evolve into defect clusters, eventually resulting in amorphization. The reduction in carrier concentration is primarily caused by irradiation-induced acceptor-like traps (mainly carbon vacancies), which capture free carriers [45]. Meanwhile, the degradation in carrier mobility arises from these defect clusters acting as scattering centers, thereby shortening the mean free path of the carriers [46]. To validate the accuracy of the fitting results, experimental Hall effect measurements were performed to quantify carrier concentration and mobility as functions of irradiation damage (dpa). As illustrated in Figure 6a,b, the Raman fitting results exhibit close consistency with the experimental data. The quantitative comparison reveals that the Pearson correlation coefficients (*r*) for carrier concentration and carrier mobility are 0.984 and 0.998, respectively, demonstrating a high degree of consistency between the two methods in evaluating radiation-induced electrical degradation.

### 3.4. The Evolution of Electrical Performance in Irradiated 4H-SiC

**Figure 7:**
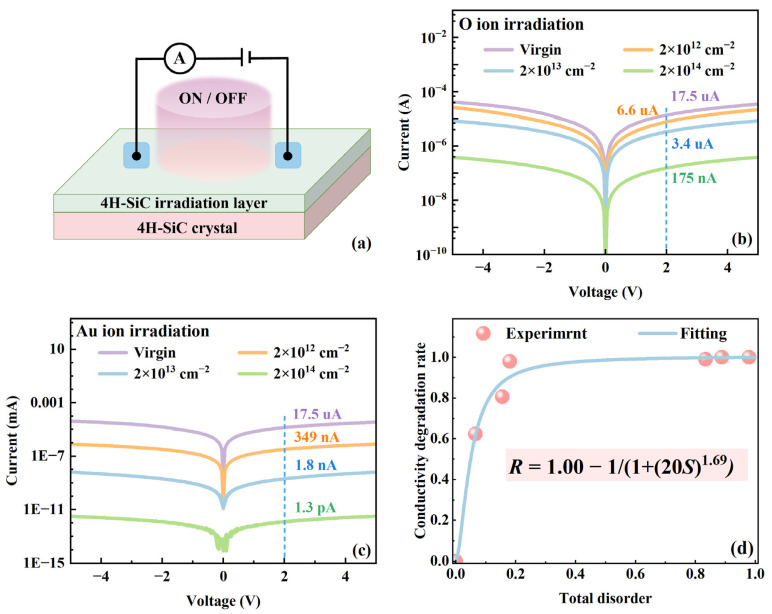
(**a**) Schematic diagram of 4H-SiC device, (**b**,**c**) I-V characteristics, (**d**) electrical conductivity degradation rate as a function of radiation damage for 4H-SiC irradiated by 2 MeV O ions and 3 MeV Au ions.

To investigate the evolution of electrical performance in irradiated 4H-SiC, a device was fabricated as illustrated in Figure 7a. The I-V characteristics are presented in Figure 7c,d, showing significant changes in current intensity after irradiation. Under O ion irradiation, the current at 2 V decreased from 17.5 μA for unirradiated 4H-SiC to 6.6 μA (a decrease of 62.3%), 3.4 μA (a decrease of 80.6%), and 175 nA (a decrease of 99.0%) for 4H-SiC irradiated with fluences of 2 × 10^12^ to 2 × 10^14^ cm^−2^, respectively. In contrast, for Au ion irradiation, the current at 2 V decreased to 349 nA (a decrease of 98.0%), 1.8 nA (a decrease of 99.1%) and 1.3 pA (a decrease of 99.8%), for fluences ranging from 2 × 10^12^ to 2 × 10^14^ cm^−2^. The conductivity degradation indicates that ion irradiation degrades the conductivity of 4H-SiC, primarily due to lattice damage that reduces both carrier concentration and mobility. Additionally, the conductivity degradation rate as a function of total disorder is plotted in Figure 7d. The conductivity degradation rate increases rapidly with rising total disorder and gradually approaches saturation, following the equation *R* = 1.00 − 1/(1 + (20*S*)^1.69^), where *S* represents the total disorder and *R* denotes the conductivity degradation rate. Specifically, “1.00” represents the ideal conductivity of a perfect lattice without any loss; “1/(1 + (20*S*)^1.69^)” is the blocking term, indicating the probability that conductivity is impeded due to irradiation-induced disorder.

## 4. Conclusions

In summary, non-destructive Raman spectroscopy, Hall effect, and current–voltage (I-V) measurements were employed to investigate the irradiation-induced damage and electrical property evolution of 4H-SiC single crystals subjected to 2 MeV O ion and 3 MeV Au ion irradiation at varying fluences. Raman spectroscopy revealed progressive amorphization and bond reorganization, with the formation of Si-Si bonds occurring prior to C-C bonds due to their lower formation energy and longer bond length. The degradation of the E_2_(TO) phonon mode and fitting results based on the DI/DS model confirmed defect-stimulated amorphization as the primary damage mechanism. Analysis of the A_1_(LO) mode, supported by Hall measurements, indicated substantial reductions in free carrier concentration and mobility, attributed to radiation-induced carrier trapping and enhanced defect scattering. These changes led to a pronounced degradation in electrical conductivity, as evidenced by I-V measurements showing a 99.8% reduction under Au ion irradiation at a fluence of 2 × 10^14^ cm^−2^. A quantitative correlation between conductivity degradation and total disorder was established, following the relation *R* = 1.00 − 1/(1 + (20*S*)^1.69^), where *R* represents the conductivity degradation rate and *S* the total disorder. This study provides a predictive framework for evaluating the evolution of SiC electrical characteristics under irradiation damage and offers critical insights for the design of radiation-hardened SiC-based devices. Temperature and energy dependency studies in subsequent work will be systematically conducted to enable more in-depth investigations aiming to uncover novel findings in the field.

## Data Availability

The original contributions presented in this study are included in the article. Further inquiries can be directed to the corresponding authors.

## Abbreviations

The following abbreviations are used in this manuscript:

SiC	silicon carbide
LOPC	longitudinal optical phonon–plasmon coupling
LO	longitudinal optical
TO	transverse optical
MCM	metal–semiconductor–metal
SRIM	Stopping and Range of Ions in Matter
I–V	current–voltage
RBS/C	Rutherford backscattering spectroscopy in channeling
TEM	transmission electron microscopy
MOCVD	metal–organic chemical vapor deposition
